# Gorlaston Cottage Hospital

**Published:** 1890-12-27

**Authors:** H. Harvey-George

**Affiliations:** Chairman. The Tower, Gorleston


### EDITOR'S LETTER-BOX.

[Our Correspondents are reminded that prolixity is a great bar to publication, and that brevity of style and concieness of statement greatly facilitate early insertion.]

### GORLESTON COTTAGE HOSPITAL.

SIR,—Will you allow me, through the means of your valuable paper, to bring before the public the great need for help required by the above useful institution[? I am told the working expenses have been kept down far below what is usually required for similar institutions, and when I state that the weekly expenses average £3 15s., it will be seen that the governors have done their utmost to work on economic principles. There are at present 75 annual subscribers, and the average annual income is £95, which leaves a deficit in the working expenses of £100. Besides this, we have been at considerable outlay in the furnishing and fittings of the new building, so that our total indebtedness now amounts to £200. This sum is so small that I feel sure that I have but to call attention to the fact, and it will be readily made up, and let me impress upon those who cannot help with their pounds to do so, at least, with their shillings, for if we can get help from the many the burden in no case will be heavy. I also ask for help with blankets and other household requirements, which are much needed in the new building. Any gifts will be most thankfully received, and the hon. secretary, Mr. Arthur William Blake, will be most happy to give any information required. I may say that up to the present the institution has afforded relief to 747 out-patients and to 48 in-patients.—I am, faithfully yours,

H. HARVEY-GEORGE, Chairman.

The Tower, Gorleston.

